# Status of cosmetic surgery and safety research: An integrative review

**DOI:** 10.1097/MD.0000000000042835

**Published:** 2025-06-13

**Authors:** Gye Gyoung Kim, Young A. Kim

**Affiliations:** a Department of Nursing, College of Life and Medical Sciences, Mokpo National University, Muan-gun, Jeollanamdo, Republic of Korea; b Department of Nursing, College of Nursing, Jeju National University, Jeju-si, Jeju-do, Republic of Korea.

**Keywords:** aesthetic surgery, cosmetic surgery, integrative review, plastic surgery, safety

## Abstract

**Background:**

Safety is a fundamental aspect of cosmetic surgery; however, the status and realities of cosmetic surgery in Korea are challenging to assess. This study reviewed the status of cosmetic surgery and safety research in Korea, a representative cosmetic surgery powerhouse in Asia.

**Methods:**

An integrative review, including both quantitative and qualitative research, was conducted on 33 papers published between 2000 and 2020.

**Results:**

Medical issues focused on the therapeutic effects of cosmetic surgery, side effects, and complications arising from cosmetic surgery. Legal issues addressed the importance of the physicians’ obligation to provide sufficient explanations for enhancing the safety of cosmetic surgery, analyzed in conjunction with legal judgments. Social issues involved the analysis of cosmetic surgery advertisements and articles, along with in-depth interviews targeting women learning about cosmetic surgery-related knowledge and information.

**Conclusion:**

Safety in cosmetic surgery should be considered essential, necessitating a thorough explanation by qualified medical professionals. Medical consumers can choose cosmetic surgery after being adequately informed of its side effects and risks. To enhance the safety of cosmetic surgery, further research that reflects the medical, legal, and social concerns is required.

## 1. Introduction

South Korea is among the most prominent countries globally where cosmetic surgery has become widespread, driven by a continuously increasing demand for such procedures.^[1]^ Although cosmetic surgery can enhance a patient’s appearance and contribute to psychological satisfaction, it also comes with potential safety concerns and associated side effects. Indeed, reports of patient fatalities and serious complications resulting from cosmetic surgery have steadily increased, bringing the intersection of cosmetic surgery and safety into focus.^[2]^ Ensuring patient safety is not only crucial but also imperative for the successful and sustained implementation of plastic surgery.^[3,4]^

Nonetheless, assessing the status and reality of cosmetic surgery in Korea is challenging, primarily because cosmetic procedures are not incorporated into essential medical practices aimed at treating and improving bodily functions. Additionally, the fact that cosmetic surgeries for aesthetic purposes are primarily performed in smaller medical institutions raises concerns regarding the vulnerability of the safety management system.^[2]^ The safety and quality maintenance of cosmetic surgery are core issues, and the accessibility of these procedures can potentially increase the associated risks. Cosmetic surgery prioritizes patient safety throughout the procedure.

Considering the global efforts for safe cosmetic surgery, France mandates a minimum waiting period of 15 days between consultation and the decision to perform the surgery.^[5]^ Australia recommends restricting surgeries and procedures for minors and advocates a cooling-off period of 3 months after consultation.^[6]^ Moreover, the regulations in Guangzhou, Taiwan, and Austria specifically govern against cosmetic surgery for adolescents.^[2]^ While efforts have been made to introduce age criteria to curb indiscriminate early cosmetic surgery in Korea, concerns about the infringement of doctors’ decision-making authority, violation of minors’ rights to choose and pursue happiness, and stigmatization of cosmetic surgery have led to the abandonment of proposed amendments to medical laws.^[7]^

The Korea Institute for Health and Medical Research has established standards and guidelines centered on efforts within medical institutions to enhance user safety during cosmetic surgeries and procedures.^[2]^ Cosmetic surgery has substantial societal implications in the long term, especially for adolescents and younger generations. However, research regarding the side effects and safety of cosmetic surgery is insufficient compared to societal interest in this field. Reviewing existing research on cosmetic surgery and safety in Korea can help strengthen the safety of patients and healthcare professionals.

Integrative reviews play a pivotal role in deriving conclusions regarding phenomena by examining their attributes or themes, thereby offering a comprehensive and in-depth understanding.^[8]^ Accordingly, this study aimed to comprehensively review the research on cosmetic surgery and safety conducted in Korea and to understand the trends and characteristics of the research. This study aimed to suggest directions for future research on cosmetic surgery and its safety.

## 2. Materials and methods

### 2.1. Study design

This study employed an integrative review method to analyze research papers related to the safety of cosmetic surgery in Korea.

### 2.2. Research procedure

The research procedure followed the 5 stages of the integrative literature review method proposed by Whittemore and Knafl^[8]^: problem identification, literature search, data evaluation, data analysis, and presentation.

#### 2.2.1. Problem identification

The research problem was framed as follows: “What is the trend of research in Korea regarding cosmetic surgery and safety?” This study aimed to comprehensively review the characteristics of recent safety-related research in cosmetic surgery in Korea to address this question.

#### 2.2.2. Literature search

##### 2.2.2.1. Keyword selection and database usage

To conduct a comprehensive search, the Korean keywords “cosmetic surgery safety,” “plastic surgery safety,” or “cosmetic procedure safety” were employed. The search was conducted using the Korean Educational and Academic Information Service and the Korean Studies Information Service System. Only papers published in academic journals were considered to ensure research quality. The selection criteria included papers written in Korean and published in Korean academic journals. Papers in which cosmetic surgery and safety were not the primary focus and papers not targeting the Korean demographic, as well as conference materials and research reports, were excluded.

#### 2.2.3. Literature search timeline

**Figure 1.**
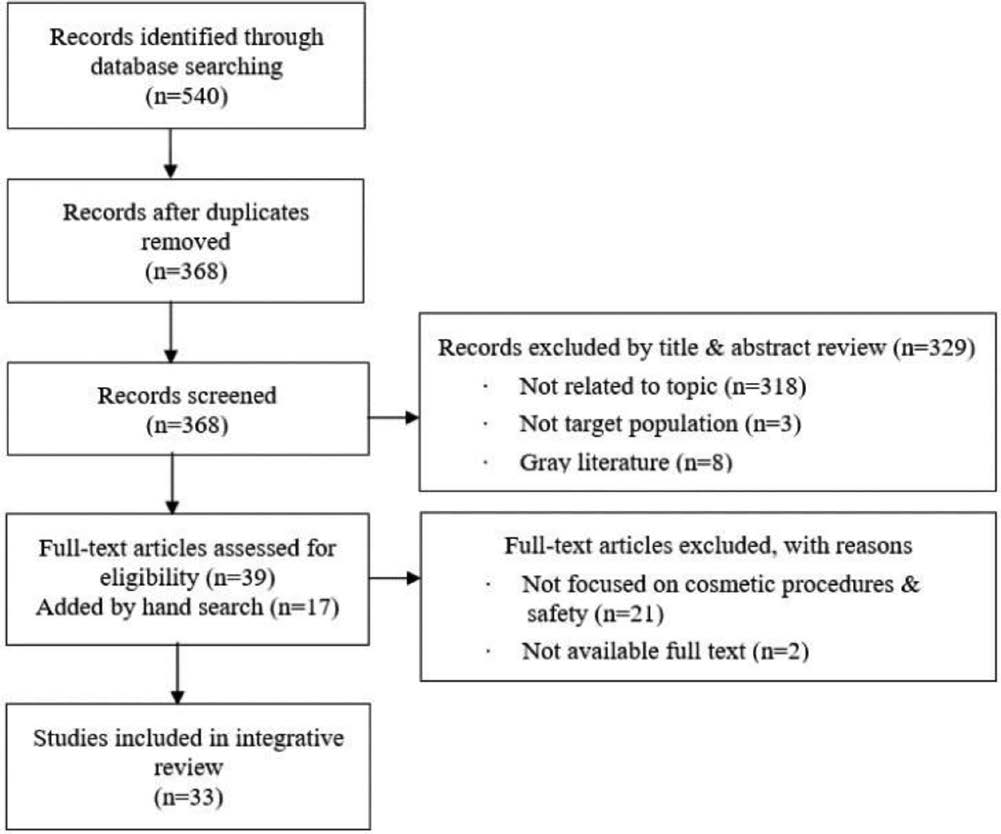
Flow chart of the study selection process.

The literature search was conducted for 1 week, starting November 2, 2021. The publication start date was not specifically designed to examine overall research trends in Korea. In total, 33 papers^[7,9–40]^ were included in this review. The participating authors rigorously applied the selection and exclusion criteria through in-depth discussions and agreement throughout the literature search (Fig. 1).

#### 2.2.4. Data evaluation

The chosen literature was categorized using Arbesman, Scheer, and Lieberman’s^[41]^ 5-level evidence model. A total of 19 case studies were classified at evidence level V, while 7 literature reviews, 3 qualitative studies, and 4 survey studies were categorized at evidence level IV. There were no studies with evidence levels of I–III (Table 1).

**Table 1:** Evidence level of the selected studies (n = 33).

Level of evidence	Research type	n (%)
I	Systematic reviews, meta-analyses, randomized controlled trials	0
II	Two groups, non-randomized	0
III	One group, non-randomized	0
IV	Descriptive studies that include analysis of outcomes	4 (12.1)
V	Case study Literature review Qualitative study	19 (57.6) 7 (21.2) 3 (9.1)

#### 2.2.5. Data analysis

To analyze the literature, a matrix was created for each paper and included the 1st author, publication year, research design, sample, academic research field, and key findings. The literature was comprehensively reviewed by categorizing it into 3 main themes: medical, social, and legal (Table 2).

**Table 2:** Descriptive summary of the selected studies (n = 33).

Category	First author (yr)	Study design	Sample (age, yr)	Academic research field (detailed)	Study topic/key findings
Medical issue (n = 21)	Han et al^[9]^	Case study	21 patients (18–52)	MP (Plastic Surgery)	Cases of ultrasound-assisted lipoplasty that reduces the possibility of bleeding and fat embolism
	Yoon et al^[10]^	Case study	1 patient (39)	MP (Neurology)	A case of acute stroke after autologous fat injection (patient death)
	Park et al^[11]^	Descriptive study	1376 medical records	MP (Otorhinolaryngology)	Complications of expanded-polytetrafluoroethylene as augmentation material in oriental patients
	Lee et al^[12]^	Case study	1 patient (48)	MP (Internal Medicine)	A case of side effects after injection of bovine collagen in the breast by an unlicensed practitioner
	Joo et al^[13]^	Case study	6 patients (21–62)	MP (Plastic Surgery)	Infection cases after augmentation rhinoplasty using a silicone implant
	Choi et al^[14]^	Case study	13 patients (28–66)	MP (Plastic Surgery)	Cases of secondary augmentation rhinoplasty with immediate autogenous dermofat graft after removal of paraffinoma
	Lee and Jeong^[15]^	Case study	132 patients (5–72)	MP (Plastic Surgery)	Cases of harvesting various kinds of grafts from the postauricular region
	Kim et al^[16]^	Case study	1 patient (20)	MP (Dermatology)	A case of delayed hypersensitivity reaction due to hyaluronic acid (Restylane^®^)
	Ahn et al^[17]^	Case study	82 patients (6–90)	MP (Plastic Surgery)	Cases of secondary adjuvant operation after free flap for functional and aesthetic purposes
	Ku et al^[18]^	Case study	1 patient (39)	MP (Ophthalmology)	A case of anterior ischemic optic neuropathy following periocular autologous fat injection
	Lee et al^[19]^	Descriptive study	12 medical records	MP (Ophthalmology)	Surgical outcome of levator recession for correction of upper eyelid retraction
	Yeo et al^[20]^	Case study	17 patients (49–73)	MP (Plastic Surgery)	Treatment cases of foreign body granuloma of the hand associated with unregulated material injection for aesthetic purpose
	Kim et al^[21]^	Case study	1 patient (28)	MP (Dermatology)	A case of multiple foreign body granuloma due to mesotherapy
	Cho et al^[22]^	Case study	1 patient (46)	MP (Ophthalmology)	A case of visual loss following injection of poly-(l)-lactic acid filler
	Yoon and Kang^[23]^	Literature review	N/A	MP (Plastic Surgery)	Perioperative considerations for patient safety in cosmetic surgery
	Kim et al^[24]^	Case study	2 patients (41)	MP (Dermatology)	Cases of cutaneous mycobacterium abscess infection after mesotherapy
	Kim et al^[25]^	Case study	1 patient (39)	MP (Internal Medicine)	A case of acute kidney injury associated with rhabdomyolysis after liposuction
	Go et al^[26]^	Case study	10 bone plates	MP (other MP)	Development of performance evaluation guidelines for bone plates in maxillofacial surgery
	Jun et al^[27]^	Case study	1 patient (15)	MP (Ophthalmology)	Corneal stromal edema during lidocaine injection for blepharoplasty
	Tang et al^[28]^	Descriptive study	124 coordinators	MP (Nursing Science)	Job analysis of cosmetic surgery medical tourism coordinators
	Seok et al^[29]^	Descriptive study	4934 safety accident cases	MP (Nursing Science)	Status of adverse events reported to the Korea Consumer Agency, focusing on harm information from 2013 to 2017
Legal issue (n = 9)	Beom^[30]^	Case study	1 court decision	SS (Law)	A judgment in violation of the doctor’s duty to explain the aftereffects of cosmetic surgery
	Mun^[31]^	Case study	1 court decision	SS (Law)	A judgment in violation of the doctor’s duty to explain the aftereffects of cosmetic surgery
	Choi^[32]^	Literature review	N/A	SS (Law)	Implications of the legal nature and responsibility for cosmetic surgery
	Jeong et al^[33]^	Case study	2 court decisions	SS (Law)	Judgment on the duty of care in explaining cosmetic surgery
	Kim^[7]^	Literature review	N/A	SS (Law)	Analysis of the Austrian law on cosmetic surgery and review implications for issues related to cosmetic surgery in South Korea
	Kwon^[34]^	Literature review	N/A	SS (Law)	Protective measures for safety rights of patients in cosmetic surgery accidents
	Yoo et al^[35]^	Literature review	N/A	MP (Ophthalmology)	Litigations in ophthalmology over 25 yr in Korea
	Kim^[36]^	Literature review	N/A	SS (Law)	Doctor’s violation of informed consent and compensation
	Baek^[37]^	Literature review	N/A	SS (Law)	Medical treatment outside the scope of the license for cosmetic surgery
Social issue (n = 3)	Lim^[38]^	Qualitative study	106 advertisements	SS (Sociology)	Advertisements that mention the science and safety of surgery to alleviate fears about cosmetic surgery
	Lim^[39]^	Qualitative study	250 articles	SS (Sociology)	Since the 1990s, articles providing cosmetic surgery information have increased, but risk information has decreased
	Tae^[40]^	Qualitative study	10 female participants (22–45)	IS (Gender Studies)	Participants preparing for cosmetic surgery and wanting to ensure the safety of cosmetic surgery and reduce uncertainty

IS = interdisciplinary studies, MP = medicine and pharmacy, N/A = not applicable, SS = social science.

#### 2.2.6. Presentation

**Figure 2.**
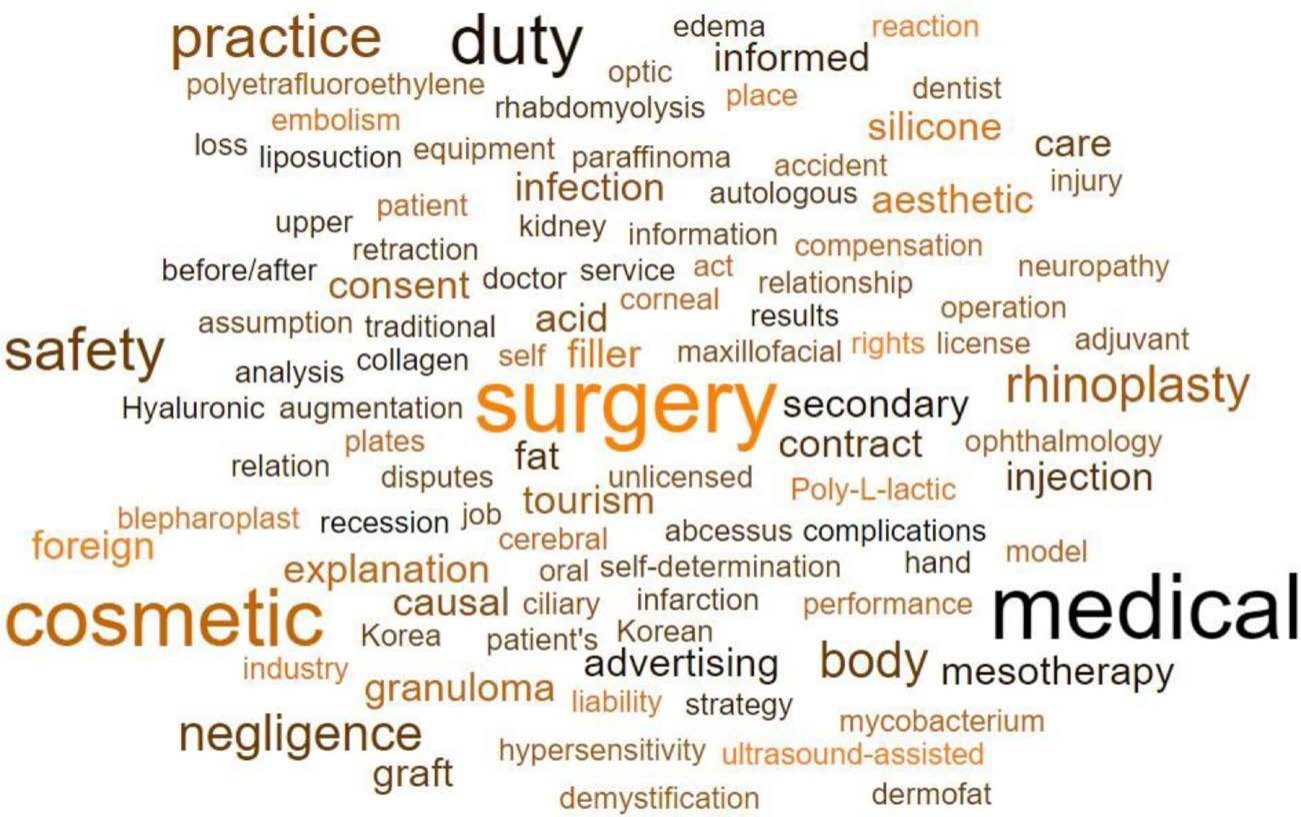
Word cloud of keywords.

The final analyzed literature was summarized, and a structured table was created (Table 2). Additionally, a word cloud was generated to visually represent the keywords (Fig. 2).

## 3. Results

### 3.1. General characteristics of the selected literature

The 33 studies selected for the final analysis on cosmetic surgery and safety were consistently published between 2000 and 2020, with a noteworthy peak in 2015 when 7 studies were published. The research designs included 19 case studies (57.6%), 7 literature reviews (21.2%), 4 survey studies (12.1%), and 3 qualitative studies (9.1%). The samples in the studies were classified as patients (17 studies), advertisements (1 study), articles (1 study), adult females (1 study), medical tourism coordinators (1 study), legal documents (3 studies), medical devices (bone plates) (1 study), and accidents (1 study). The research fields included medicine and pharmacy (22 studies), social sciences (SS; 10 studies), and interdisciplinary (1 study). Research trends were analyzed by dividing them into 3 major categories (Table 2).

### 3.2. Medical issues

A total of 21 studies (63.6%) were in the medical issues category. The following is an examination of studies based on research design types.

There were a total of 16 case studies. The samples included patients from 15 studies and medical devices from 1 study. Patient-focused studies have addressed cases related to illegal injection treatment for cosmetic purposes,^[12,14,20]^ therapeutic effects,^[9,15,17]^ and side effects^[10,13,16,18,21,22,24–27]^ resulting from cosmetic surgery or procedures. Side effects range from local inflammatory reactions in the cosmetic surgery area to various serious consequences, for example, death following acute cerebral infarction. A study on medical devices^[26]^ developed guidelines for evaluating the performance of maxillofacial fixation plates to enhance the quality of medical devices. All studies in the academic research field were classified under “medical and pharmacy,” specifically in “plastic surgery,”^[9,13–15,17,20]^ “ophthalmology,”^[18,22,27]^ “dermatology,”^[16,21,24]^ “internal medicine,”^[12,25]^ “neurology,”^[10]^ and “other medicine and pharmacy.”^[26]^

There were 4 descriptive studies. The samples included medical records in 2 studies, medical tourism coordinators in 1 study, and safety accident reporting data in 1 study. These studies retrospectively analyzed patient medical records over several years to report the therapeutic effects of cosmetic surgery,^[11,19]^ assessed the duties of medical tourism coordinators working in plastic surgery,^[28]^ and evaluated the status of accidents reported to the Korea Consumer Agency due to medical issues.^[29]^ All studies in the academic research field were in the “medical and pharmacy” field, with specific categorizations in “nursing science,”^[28,29]^ “ophthalmology,”^[19]^ and “otorhinolaryngology.”^[11]^

Finally, 1 literature review addressed the considerations for pre- and postsurgical patient safety in plastic surgery. This study belonged to the “medical and pharmacy” field, specifically “plastic surgery.”^[23]^

### 3.3. Legal issues

A total of 9 (27.3%) studies were categorized under legal issues and had 2 design types.

There were 6 literature reviews. These reviews covered the legal nature and responsibility of plastic surgery,^[32]^ legal protection and legislative considerations for plastic surgery,^[7]^ protection of the safety rights of patients involved in accidents related to cosmetic surgery,^[34]^ medical lawsuits related to ophthalmology for over 25 years,^[35]^ damages for violation of a doctor’s duty to explain,^[37]^ and a review of licensure and medical practices beyond licensing for cosmetic surgery.^[38]^ The academic research fields for these studies included 5 in the “SS” field under “law”^[32–34,37,38]^ and 1 in the “medical and pharmacy” field under “ophthalmology.”^[35]^

There were also 3 case studies. The samples in these studies were 1 to 2 legal judgment documents. Studies have analyzed legal judgments related to violations of a doctor’s duty to explain the sequelae of cosmetic surgery^[30,31]^ and legal judgments related to the duty of care in explaining plastic surgery.^[36]^ All 3 studies fell under the academic research field of “SS” under “law.”

### 3.4. Social issues

A total of 3 (9.1%) papers were categorized under social issues, and all were qualitative studies. The sample included monthly cosmetic surgery advertisements,^[38]^ newspapers and dailies,^[39]^ and adult female participants.^[40]^ Cosmetic surgery advertisements emphasized the scientific and safety aspects of surgery to alleviate fear,^[38]^ and articles on cosmetic surgery suggested the need for sufficient risk disclosure.^[39]^ Female participants sought knowledge and information from online cosmetic surgery-related communities as part of their preparation for cosmetic surgery. This served the dual purpose of ensuring safety and reducing uncertainty.^[40]^

## 4. Discussion

This study delves into the landscape of Korean research on cosmetic surgery and safety, aiming for a comprehensive understanding of its trends and characteristics. By meticulously reviewing existing studies, it sought to shed light on current knowledge and pave the way for future advancements. We found that the trends in research on cosmetic surgery and safety in South Korea, across various aspects, can be categorized into 3 main groups. Publications on medical issues accounted for more than half, surpassing those on legal and social issues.

Studies focusing on the safety, therapeutic effects, and side effects of cosmetic surgical procedures have gained prominence in the medical domain. These studies emphasize the importance of verifying the safety of existing treatments and materials, as well as sharing cases involving therapeutic and side effects. Such practices are essential to prevent unnecessary complications, enhance patient satisfaction, and improve surgical outcomes.^[3]^ To maintain safety and make informed choices, patients, the general public, and plastic surgery medical professionals should continually assess the stability of cosmetic surgery in line with evolving data.^[4]^ Identifying risk factors and disseminating information regarding complications specific to each procedure can facilitate patient choice and education, especially in response to the growing demand for cosmetic surgery.^[42]^ Conversely, research on psychological aspects is challenging. Considering that patients attempting cosmetic procedures for aesthetic purposes tend to have statistically higher depression scores than the general population,^[43,44]^ it is desirable to undertake studies that consider psychological aspects in the field of cosmetic surgery and safety.

In the legal context, cosmetic surgery should expand the scope of disclosure obligations compared to general medical practices. Being vigilant about diagnostic accuracy can enhance both patient and physician safety, and potentially reduce medical lawsuits. Obtaining valid and information-based consent is crucial for legitimizing medical-surgical procedures.^[45]^ Cosmetic surgery poses a considerably high risk of medical malpractice claims, with most claims arising from inappropriate patient selection criteria and a lack of communication between patients and physicians.^[46]^ Ethical aspects such as patient autonomy, prior consent, good intentions, and prohibition of illegal practices require careful consideration.^[47]^ Taking these factors into account can promote the safety of both patients and physicians and yield positive outcomes.

From a social perspective, advertisements and articles highlighting the scientific and safety aspects of cosmetic surgery rather than its risks were prevalent. It was discovered that individuals often turn to online cosmetic surgery communities to gather information to ensure the safety of cosmetic surgery and reduce uncertainty. In the 21st century, the media has played a considerable role in shaping societal beauty standards.^[48]^ The Internet and social media are increasingly influencing and playing crucial roles in cosmetic surgery. Consequently, patients have greater access to information, leading to the risk of fostering unrealistic expectations.^[49]^ The influence of cosmetic surgery on society appears to be expanding. Nevertheless, owing to limited research and the dearth of more recent literature on cosmetic surgery compared with research on other issues, there is a pressing need for additional studies that reflect current societal trends.

The primary academic research area of the selected literature was medicine, with research also being conducted in law, sociology, and other fields. The increasing participation in academic research in fields other than mainstream medical, legal, and social issues is particularly noteworthy. For example, disciplines such as nursing contributed to addressing medical issues, while medicine played a role in addressing legal issues, and women’s studies were involved in examining social issues. Considering the growing social interest and occurrence of safety issues, collaborative efforts across various academic research fields are necessary to improve cosmetic surgery and safety issues. Medical and plastic surgery technologies continue to advance, and the introduction of new medical technologies and materials indicates the need for research from the perspectives of safety and effectiveness. Research aimed at developing safer and more effective surgical methods can improve patient outcomes. Furthermore, despite the gradual decrease in the age of cosmetic surgery consumers,^[1,48,50]^ research findings pertaining to adolescents and younger generations seeking cosmetic surgery are scarce. Consequently, there is a pressing need for studies investigating the side effects and safety issues associated with cosmetic surgery in teenagers and young adults.

It is important to note that this study was based on academic papers presented in Korean, which limits the generalizability of the research results. Expanding the search scope could potentially uncover additional relevant studies. It is worth acknowledging that the reliance of relevant studies on keyword-based identification may have constrained the literature search. Finally, similar to other reviews, it cannot be presumed that all pertinent papers of interest were successfully identified during the search and selection procedures.

Despite these limitations, this study is expected to provide evidence-based information on cosmetic surgery safety by integrating the results of other studies. It is anticipated to benefit various stakeholders, including consumers of cosmetic surgery, medical professionals, and companies. This study serves as a foundation for building a safer and more trustworthy market for cosmetic surgery.

## 5. Conclusions

Research on cosmetic surgery and safety in Korea has been conducted in the medical, legal, and social domains. Considering the rising demand for cosmetic surgery and the ongoing advancements in technology and materials, research findings must be viewed within the context of this demand and the potential for future developments. Considering the importance and safety of cosmetic surgery, ongoing efforts are needed to expand the scope of research participants and topics in future studies.

## Author contributions

**Formal analysis:** Gye Gyoung Kim, Young A. Kim.

**Conceptualization:** Young A. Kim.

**Data curation:** Young A. Kim.

**Investigation:** Young A. Kim.

**Methodology:** Young A. Kim.

**Supervision:** Young A. Kim.

**Visualization:** Young A. Kim.

**Validation:** Gye Gyoung Kim, Young A. Kim.

**Writing – original draft:** Young A. Kim.

**Writing – review & editing:** Gye Gyoung Kim, Young A. Kim.
